# Post-partum tubal ligation at time of cesarean delivery or via laparoscopy as an interval sterilization has similar effects on ovarian reserve

**DOI:** 10.4274/jtgga.galenos.2018.2018.0087

**Published:** 2020-03-06

**Authors:** Ali Gemici, Yavuz Emre Şükür, Fırat Tülek, Salih Taşkın, Cem Somer Atabekoğlu

**Affiliations:** 1 Ankara University Faculty of Medicine, Department of Obstetrics and Gynecology, Ankara, Turkey

**Keywords:** Tubal ligation, ovarian reserve, anti-müllerian hormone, cesarean section, laparoscopy

## Abstract

**Objective:**

To observe and compare the effect of postpartum tubal ligation (TL) procedures on ovarian reserve at women desiring TL as a contraceptive method at the end of pregnancy.

**Material and Methods:**

Eighty-one women were included in the prospective study. TL was performed at the time of cesarean delivery (CD) (n=49) and as an interval procedure by laparoscopy (LS) in the postpartum period (n=32). Anti-müllerian hormone (AMH) was used to determine ovarian reserve. Blood samples were taken twice from each subject; the first sample was taken before delivery from all subjects and the second sample was taken 4 months after sterilization. AMH level differences were compared in each group and between groups.

**Results:**

The preoperative AMH values of CD and LS groups were similar 2.30 (maximum: 5.20, minimum: 0.42) ng/mL and 1.80 (maximum: 3.50, minimum: 0.40) ng/mL, respectively (p=0.262). The postoperative AMH values of the CD and LS groups were 1.30 (maximum: 2.60, minimum: 0.30) ng/mL and 0.90 (maximum: 2.50, minimum: 0.20) ng/mL, respectively (p=0.284). When the preoperative and postoperative values of each group were compared the change was statistically significant for both groups p<0.001. The decrease in mean AMH values in the CD and LS groups were 37.83% and 44.15%, respectively. The percentage changes of AMH values were not statistically significant (p=0.286).

**Conclusion:**

TL at the time of CD and interval sterilization with LS have similar effects on ovarian reserve.

## Introduction

Tubal ligation (TL) is a permanent contraceptive method preferred mostly by women with more than one child (1). It is known to be safe procedure but utero-ovarian blood flow disruption has been considered as an adverse effect of the procedure (2,3,4,5,6). The debate on this issue has continued since the 1950s (2). Several studies were designed to observe the effect of TL on ovarian function (3,4,5,6,7,8,9,11,12,13,14,15,16,17,18,19,20,21). The first studies were primarily based on observational data (3,4,5,6). It has been reported that TL may result in menstrual irregularity, mainly shortening of menstrual bleeding due to disruption of ovarian function (3,4). However, all previous studies were observational and needed to be checked by objective measurements.

For the last decades, more objective parameters were used to measure ovarian reserve and function; namely, follicle-stimulating hormone, luteinizing hormone, estradiol (E2), anti-müllerian hormone (AMH), and ultrasound (Doppler blood flow and antral follicle count) for determining the effect of TL (9,11,12,13,14,15,16,17,18,19,20,21,22). Until recently, AMH was mainly used to assess ovarian reserve status (21,22,23).

Controversial results regarding the effect of salpingectomy on ovarian reserve measured by AMH have been reported (7,8). Ye et al. (7) reported decreased ovarian reserve after salpingectomy. However, Venturella et al. (8) reported that surgical excision including the removal of mesosalpinx with salpingectomy did not cause any negative effect on ovarian reserve. Bipolar coagulation was thought to create less tissue damage; however, laparoscopic TL using bipolar electrocoagulation was reported to cause lower AMH values (11,12).

It is certainly the case that most women usually express their desire for TL in their pregnancy period (24). In the case of TL at the time of cesarean delivery (CD), there must be an indication for the CD. However, to the dissatisfaction of some patients, physicians may be reluctant to proceed with cesarean delivery for the sole purpose of sterilization; these patients are referred for interval sterilization with laparoscopy (LS).

In the present retrospective cohort study, we aimed to compare the effects of two different postpartum TL modalities on ovarian reserve in women desiring TL at their pregnancy period; TL at the time of CD and interval sterilization with LS.

## Material and Methods

Patients who had a desire for TL during pregnancy at the obstetrics and gynecology clinics of a university hospital between November 2011 and May 2013 were enrolled in this retrospective cohort study. The study was approved by the Institutional Review Board (IRB approval no: 16-624-13, date: 11/11/2013).

### Patient selection

The first study group consisted of women who underwent TL during a scheduled or emergency CD. The second study group consisted of women who underwent laparoscopic TL in the postpartum period, 2-4 months after vaginal delivery (interval sterilization). The inclusion criteria were women aged between 30 and 40 years who had a desire for TL at the end of their current pregnancy. The exclusion criteria were previous ovarian surgery, polycystic ovary syndrome, any type of cancer and/or pelvic radiotherapy history, and any medication affecting ovarian response (oral contraceptive pills, danazol, gonadotropin-releasing hormone analogues).

**Figure 1:**
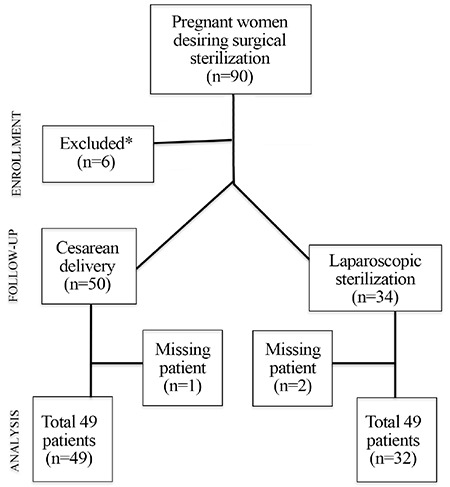
Flow-chart of patients *Four of the excluded patients had polycystic ovaries and two had previous ovarian surgery because of endometrioma

A total of 90 patients were assessed for eligibility. After application of the inclusion and exclusion criteria, 84 patients were found to be eligible. Four of the excluded patients had polycystic ovaries and two had previous ovarian surgery because of endometrioma. Three more patients, one from the CD group and two from the laparoscopic TL group were lost during the follow-up period. As a result, 49 patients from the first group and 32 patients from the second group were included in the final analyses. Figure 1 shows the flow-chart of patients who were assessed, excluded, and followed up.

### Surgical procedures

In our clinic, the Pomeroy technique is preferred for TL during CD. In this technique, the fallopian tube is inspected and held from the isthmic portion, then ligated using a number 0 absorbable suture by penetrating the avascular part of the mesosalpinx. The upper part of the ligated portion is cut with scissors and the remaining ligated part is checked for hemostasis. Then, the same procedure is repeated for the contralateral fallopian tube (25). Laparoscopic TL is performed under general anesthesia with the insertion of two to three ports. First, the pelvic anatomy is inspected. Then the fallopian tube is held from the avascular isthmic portion and cauterized using bipolar forceps until it becomes white, and subsequently cut with scissors without using electro-cautery. The cut portion is checked for hemostasis. The same procedure is repeated for the contralateral fallopian tube. There is no intervention with the ovaries during either of the procedures.

### Main outcome parameters

The main outcome measure was preoperative and postoperative AMH changes. The percentage change between preoperative and postoperative AMH values were calculated for both groups for intergroup comparison.

### Anti-müllerian hormone assay

The first blood samples were collected in the third trimester for each group when pre-labor assessment took place. The second blood samples were collected four months after sterilization. Blood samples were centrifuged at 4000 rpm for 10 minutes and stored at -80 °C until required for analysis. Upon collection of all samples, serum AMH levels were determined on the same day by using a commercially available enzyme-linked immune sorbent assay kit (Beckman Coulter Inc., Paris, France) with a lowest detection limit of 0.14 ng/mL. Intra- and inter-assay coefficients of variation were 12.3% and 14.2%, respectively.

### Statistical analysis

Data analyses were performed using the SPSS Version 21.0 statistical software package (IBM Corporation, Armonk, NYC, USA). Samples were tested using Kolmogorov-Smirnov test to determine normality of distributions. Continuous variables were compared using the Mann-Whitney U test according to the distribution of each variable. The Wilcoxon signed-rank test was used for the comparison of preoperative and postoperative AMH values. A p value of <0.05 was considered statistically significant. In determining the difference of two methods by time, a p value of <0.025 was considered statistically significant, according to Bonferroni’s correction.

## Results

A total of 81 participants were evaluated, 49 patients underwent TL during CD, and 32 patients underwent TL with LS. The mean age of the CD group and LS group was 34.4±2.25 and 35.1±2.27 years, respectively. The median parity value of both groups was 3. The body mass index (BMI) of the CD group and LS group was 28.8 and 29.1 kg/m2, respectively. Both groups were similar with consideration to age, parity, BMI, and live birth numbers (Table 1). The median preoperative AMH value for the CD group was 2.30 (maximum: 5.20, minimum: 0.42) ng/dL and 1.80 (maximum: 3.50, minimum: 0.40) ng/dL for the LS group. Both groups were similar according to preoperative median AMH values (p=0.262). The median postoperative AMH value for the CD group was 1.30 (maximum: 2.60, minimum: 0.30) ng/dL and 0.90 (maximum: 2.50, minimum: 0.20) ng/dL for the LS group. Both groups were similar according to the median postoperative AMH values (p=0.284). The median postoperative AMH values of both groups were lower than the preoperative median AMH values; this difference was statistically significant for both groups (p<0.001). The percentage change of the median AMH value of the CD group and LS group was 37.83% and 44.15%, respectively. The percentage change of the AMH values was similar between the groups (p=0.286) (Table 2).

## Discussion

The present study was conducted to compare the effects of two different postpartum TL methods on ovarian reserve. According to our results, both TL at the time of CD and interval TL with LS were detected to significantly decrease serum AMH levels. However, when preoperative and postoperative percentage changes were compared between the groups, similar changes were detected.

The speculated mechanism of the adverse effect of TL on ovaries was blood perfusion disturbances (2,3,4,5,6). However, studies evaluating utero-ovarian Doppler blood flow changes were unable to demonstrate any difference, either on blood perfusion or follicular phase hormonal values after TL (12,13,14,15,16,17,18). Nevertheless, two studies demonstrated decreased mid-luteal progesterone levels contributing menstrual disturbances after TL (13,14). Surgical sterilization can result in subtle changes in ovarian function, even though ovulation itself is not affected (16,17). Kelekci et al. (18) and Kutlar et al. (19) demonstrated increased resistivity index of utero-ovarian blood flow without statistical significance. In addition, the negative impact of TL on ovarian reserve was histologically confirmed in an animal study (20). In a recent study that compared the effect of TL and salpingectomy at the time of CD on ovarian reserve, similar AMH changes were detected 6-8 weeks postoperatively (21).

In this study, we detected statistically decreased postoperative AMH levels in both procedures. The percentage change of AMH was lower in the CD group, but we failed to demonstrate a significant difference between the groups.

The mesosalpinx is one of the blood perfusion sources for ovaries (2). When tubal patency is disrupted for sterilization, blood perfusion on the mesosalpinx may be intervened (1,2). Salpingectomy is an invasive procedure compared with TL. Ganer Herman et al. (21) reported that even salpingectomy during CD resulted with similar AMH changes when compared with TL performed during CD. In the present study, the decrease of AMH percentage was lower in the CD group, but this change was not statistically significant. The intervention of the avascular mesosalpingeal part of the uterus may be simpler compared with interval sterilization through LS. This might be a reason for this percentage difference.

Previously, Ozyer et al. (22) compared AMH values in women who underwent TL at the time of CD and TL with mini-laparotomy approximately 1 year after surgery. They found higher AMH levels in women who underwent TL at the time of CD. Interestingly, different from Ozyer et al. (22), postoperative AMH values in both groups were prominently decreased in the present study. This may because of our study population. Our study cohort was constituted by pregnant women and women in the early postpartum period, as the major strength of this study. None of the patients started to menstruate during the study period. The sharp fall of AMH could be a result of the inability to create a new AMH secreting follicle cohort after TL. In the long term, AMH levels may probably be compensated after ovarian function recruitment. The classic dogma regarding ovarian physiology dictates that the number of primordial follicles is constant and cannot be replenished. AMH is expressed by granulosa cells; its expression is initiated in the smallest growing follicles and declines in the early antral stages as one follicle is selected for dominance and the rest become atretic. Sönmezer et al. (23) proposed a theory of a compensatory mechanism following surgical ovarian damage. It is possible that any acute damage may stimulate more primordial follicles from the stockpile start to growing (23). This may be possible without any inhibitory situation such as pregnancy and lactation. In our study, there was no compensatory mechanism because all patients were pregnant or in the early postpartum period, and the sharp decrease might be due to the absence of this compensatory mechanism.

The other strength of our study was the comparison of the effects of the two most frequently used TL methods by assessing preoperative and postoperative AMH values. To the best of our knowledge, this is the first survey to compare two different TL procedures and is therefore a pioneer for similar studies.

### Study limitations

The long-term postoperative values were not evaluated in our study. Also, cesarean section itself might have a negative effect on ovarian reserve, which could not be determined in our study. The effect of pregnancy on the bioactivity of AMH is still unknown. There are studies stating that AMH is invariant during the pregnancy period (9,26). In contrast, some studies reported a decrease of circulating AMH levels during the pregnancy period, but the observations are inconsistent (27,28). It could be better if the ovarian reserve markers were assessed before the pregnancy period, but it could only be possible for intrauterine insemination or in vitro fertilization patients who we may not be offered a surgical contraceptive technique in the postpartum period. Another limitation of our study was the low number of patients in the study groups. Moreover, the lack of a power analysis before data review can be noted as a limitation.

In conclusion, this study showed that both management techniques for TL had similar but negative impacts on ovarian reserve. These data can be beneficial for both patients and physicians, that the timing of TL may not have different effects on ovarian reserve. However, further studies are needed with larger cohorts evaluating long-term AMH values, particularly after recovery of normal menstrual periods.

## Figures and Tables

**Table 1:**
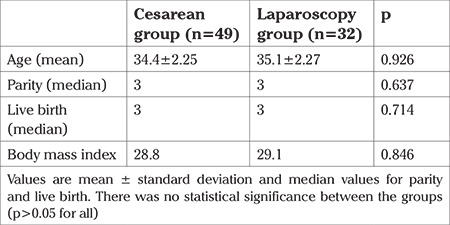
Patient characteristics

**Table 2:**
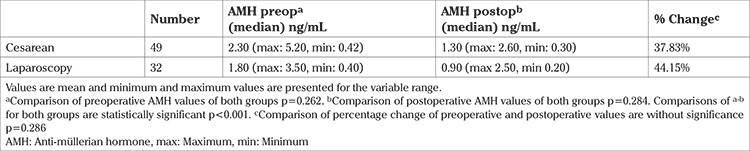
Change of the anti-müllerian hormone values for both groups
